# Medium-term outcomes of myocarditis and pericarditis following BNT162b2 vaccination among adolescents in Hong Kong

**DOI:** 10.1080/22221751.2022.2128436

**Published:** 2022-10-26

**Authors:** Gilbert T. Chua, Sabrina Tsao, Mike Yat Wah Kwan, Tak Ching Yu, Agnes Sze Yin Leung, Ka Wah Li, Calvin Chit Kwong Chow, Wai Hung Ku, Janette Kwok, Kelvin K. W. To, Yu Lung Lau, Francisco Tsz Tsun Lai, Ian Chi Kei Wong, Patrick Ip

**Affiliations:** a Department of Paediatrics and Adolescent Medicine, Li Ka Shing Faculty of Medicine, The University of Hong Kong, Hong Kong SAR, People’s Republic of China; b Department of Paediatrics and Adolescent Medicine, The Hong Kong Children’s Hospital, Hong Kong SAR, People’s Republic of China; c Department of Paediatrics and Adolescent Medicine, Queen Mary Hospital, Hong Kong SAR, People’s Republic of China; d Department of Paediatrics and Adolescent Medicine, Princess Margaret Hospital, Hong Kong SAR, People’s Republic of China; e Department of Paediatrics and Adolescent Medicine, Pamela Youde Nethersole Eastern Hospital, Hong Kong SAR, People’s Republic of China; f Department of Pediatrics, Prince of Wales Hospital, The Chinese University of Hong Kong, Hong Kong SAR, People’s Republic of China; g Department of Paediatrics and Adolescent Medicine, Tuen Mun Hospital, Hong Kong SAR, People’s Republic of China; h Department of Paediatrics and Adolescent Medicine, United Christian Hospital, Hong Kong SAR, People’s Republic of China; i Department of Paediatrics and Adolescent Medicine, Tseng Kwan O Hospital, Hong Kong SAR, People’s Republic of China; j Division of Transplantation and Immunogenetics, Department of Pathology, Queen Mary Hospital, Hong Kong SAR, People’s Republic of China; k Department of Microbiology, Carol Yu Centre for Infection, Li Ka Shing Faculty of Medicine, School of Clinical Medicine, The University of Hong Kong, Hong Kong SAR, People’s Republic of China; l Centre for Safe Medication Practice and Research, Department of Pharmacology and Pharmacy, The University of Hong Kong, Hong Kong SAR, People’s Republic of China; m Research Department of Practice and Policy, UCL School of Pharmacy, University College, London, United Kingdom

**Keywords:** Adolescents, BNT162b2, COVID-19, medium-term, myocarditis

## Abstract

In this study, we examined the clinical and electrophysiological outcomes of adolescents in Hong Kong who developed myocarditis or pericarditis following BNT162b2 vaccination for COVID-19, and followed-up for 60–180 days after their initial diagnosis. Clinical assessments included electrocardiogram (ECG) and echocardiogram at the initial admission and follow-up were compared. Treadmill testing was also performed in some cases. Between 14 June 2021 and 16 February 2022, 53 subjects were approached to participate in this follow-up study, of which 28 patients were followed up for >60 days with a median follow-up period of 100 days (range, 61–178 days) and were included in this study. On admission, 23 patients had ECG abnormalities but no high-grade atrioventricular block. Six patients had echocardiogram abnormalities, including reduced contractility, small rim pericardial effusions, and hyperechoic ventricular walls. All patients achieved complete recovery on follow-up. After discharge, 10 patients (35.7%) reported symptoms, including occasional chest pain, shortness of breath, reduced exercise tolerance, and recurrent vasovagal near-syncope. At follow-up, assessments, including ECGs, were almost all normal. Among the three patients with possible ECG abnormalities, all their echocardiograms or treadmill testings were normal. Sixteen patients (57.1%) underwent treadmill testing at a median of 117 days post-admission, which were also normal. However, at follow-up, there was a significant mean bodyweight increase of 1.81 kg (95%CI 0.47-3.1 kg, *p* = 0.01), possibly due to exercise restriction. In conclusion, most adolescents experiencing myocarditis and pericarditis following BNT162b2 vaccination achieved complete recovery. Some patients developed non-specific persistent symptoms, and bodyweight changes shall be monitored.

## Introduction

The BNT162b2 vaccine was demonstrated to be largely safe and effective against COVID-19 in clinical trials.[1,2] However, following the widespread use of the BNT162b2 vaccine worldwide, numerous reports of myocarditis and pericarditis, particularly in male adolescents receiving their second dose 21 days after the first.[3–5] In Hong Kong, our pharmacovigilance population-based cohort study showed that all patients had mild symptoms during their acute admission and did not require inotropic, ventilatory, and circulatory support, [6] which largely agreed with the most recent evidence on the mild clinical presentation during the hospital course and short-term outcomes in adolescents and children. [7–13] The United States Centers for Disease Control and Prevention (CDC) have also surveyed COVID-19 patients and their clinicians to understand the long-term health effects of myocarditis following mRNA vaccination.[14] However, data on the medium-term clinical outcomes at follow-up is lacking. Here, we describe the medium-term (60–180 days) clinical and non-invasive electrophysiological outcomes of Hong Kong adolescents diagnosed with myocarditis following BNT162b2 vaccination.

## Methods

This was an observational follow-up study of the population-based vaccine safety monitoring program initiated by the Hong Kong regulatory authority.[6] Following BNT162b2 vaccination, all children diagnosed with myocarditis or pericarditis according to the definitions for myocarditis and pericarditis of the Cardiovascular Injury-Coalition for Epidemic Preparedness Innovations (CEPI) and the Brighton Working Group were followed up.[15] These patients were referred from all regional hospitals to a centralized clinic set up at the Hong Kong Children’s Hospital for follow-up and counseling. Visits to the clinic were arranged within 12 weeks of the patient’s initial admission. A pediatric cardiologist (ST) and two pediatric immunologists (GC and MK) provided the follow-up assessments.

The inclusion criteria for participation in this study were: 1) adolescents between 12 and 18 years of age at the time of diagnosis, 2) follow-up was between 60 and 180 days from the initial diagnosis, and 3) written consent was provided. The exclusion criteria were: 1) > 18 or <12 years of age at the time of diagnosis, 2) received COVID-19 vaccines other than the BNT162b2 vaccine, 3) had an alternative cause such as viral infection, 4) follow-up was <60 or >180 days from the initial diagnosis, and 5) did not provide consent for the study.

Serial ECGs, serial echocardiograms, and cardiac magnetic resonance imaging (cMRI) performed during acute admission and follow-up at the referring hospitals were reviewed by a single investigator (ST). All ECGs were performed at a speed of 25 mm/second and 10 mm/mV gain. Reviewed parameters included heart rate, QRS axis, PR interval, QRS duration, QT and QTc intervals, ST/T wave abnormalities, including T wave inversion (excluding aVR and V1-V2) and biphasic or flat T waves, and QRS voltages. Treadmill testing conducted around three months after discharge was only available at some centers due to the limited resources during the COVID-19 pandemic. All subjects were advised to avoid strenuous exercise for three months after discharge or until treadmill testing was normal.

Clinical symptoms and bodyweight changes between acute admission and follow-up were recorded and reviewed. One sample T-test was used to determine any significant changes in the bodyweight, and a *p*-value of <0.05 is considered statistically significant. Assessments included serial ECGs, serial echocardiograms, cMRI, and treadmill testing.

This study was approved by the Institutional Review Board of the University of Hong Kong/Hospital Authority Hong Kong West Cluster (Reference: UW 21-548), the Hospital Authority Central Institutional Review Board (CIRB-2021-003-4), and the Department of Health Ethics Committee (LM21/2021). Written consent and assent were obtained from the parents and participating patients, respectively.

## Results

Between 14 June 2021 and 16 February 2022, 53 adolescents who had received the BNT162b2 vaccination were diagnosed with myocarditis or pericarditis according to the CEPI and Brighton Working Group Criteria. Among these patients, 28 fulfilled the eligibility criteria and consented to participate in this study. Table 1 gives a summary of the patient characteristics, which showed 17 (60.7%) had myocarditis and 11 (39.3%) had perimyocarditis, of which 17 (60.7%) were definite cases, seven (25.0%) were probable cases, and four (14.3%) were possible cases. The median follow-up period was 100 days (range from 61 to 178 days). Other potential viral infections, including SARS-CoV-2 infection, were excluded as possible causes of myocarditis and pericarditis. Details of the investigations have been published elsewhere.[6]

**Table 1.** Patient characteristics and investigations.

Pt no	Age/Sex	Dx	Level of certainty	Duration of Follow-up (days)	First or second dose?	BW Change (kg)	Symptoms on follow-up	Peak Troponin (hsTnI/hsTnT/TnI/TnT)* (ng/L)	cMRI findings at diagnosis	ECG during admission	Follow-up ECG	Echocardiogram during admission	Follow-up Echocardiogram	Treadmill testing
1	15/M	Perimyocarditis	Definite	95	Second	0.5	Asymptomatic	Peak TnI 2506 TnT 793	Normal biV fx, no PEF, patchy edema, diffuse EGE and patchy pericardial/subepicardial LGE	Transient 1st degree AVB; 3 mm STE in V3-V4	Normal	Normal fx. Incidental finding of small coronary fistula to pulmonary trunk	N/A	Normal
2	14/M	Myocarditis	Definite	66	Second	−1.0	Asymptomatic	TnI 6342 TnT 646	Evidence of myocarditis. Normal RV fx (RVEF 50.6%), borderline LV fx (LVEF 51.5%). RLPV narrowing.	1st degree AVB; TWI III, avF; biphasic Ts V4-V6	N/A	Admission: borderline LV function, mild septal hypokinesia. Repeat echo pre-discharge: Satisfactory biventricular function	N/A	Normal
3	13/M	Perimyocarditis	Definite	88	Second	−0.2	Asymptomatic	TnT 1749	Normal biV volume, borderline biV fx (LVEF 52%, RVEF 46%). Evidence of perimyocarditis with LGE	2–4 mm STE in V3, V4, V6; STD in aVR, III, V1	Normal	Normal	N/A	Normal
4	13/F	Perimyocarditis	Definite	146	First	5.1	Asymptomatic	TnI 2974 TnT 302	Evidence of perimyocarditis. Borderline biV function (LVEF 52%, RVEF 40%)	2 mm in V5-V6; 1 mm STD in aVR, TWI in III	Normal	Normal	N/A	Normal
5	16/M	Perimyocarditis	Definite	106	Second	2.1	Admitted for severe chest pain 12 weeks after the initial admission, but normal ECG and Troponin	TnI 2882, TnT 948	Evidence of perimyocarditis with Edema, hyperemia and LGE. Small PEF. Borderline biV fx (LVEF 52%, RVEF 42%)	2–5 mm STE V2-V6; TWI aVL	Possible LVH, 2 mm STE in V3-V4	Normal LV function. Mildly impaired LV global longitudinal strain – 17%	N/A	Normal
6	15/M	Myocarditis	Definite	149	Second	N/A	Asymptomatic	hsTnI 11415	Normal biV fx. Evidence of myocarditis	Isolated PVC	Normal	Normal	N/A	Normal
7	15/M	Perimyocarditis	Definite	148	Second	−0.2	Asymptomatic	hsTnI 18507	LVEF 54.4%, increased STIR signal in the myocardium, faint patchy LGE, trace PEF	2 mm STE II, III, aVF, V5-V6; 2 mm STD V1-V2; Coved ST with TWI II, III, aVF, V4-V6	Normal	Borderline LV fx LVFS 28%, minimal pericardial effusion. Pre-discharge echocardiogram: normal LV fx LVFS 41%, small effusion	Normal	Normal
8	17/M	Perimyocarditis	Definite	178	First	1.1	Exercise-induced shortness of breath and tiredness from baseline 30 min to 10 min	hsTnI 19110	Evidence of myocarditis. LVEF 62%, RVEF 56%	1st degree AVB; Isolated PVC; 2 mm STE V2-V5	Normal	Tiny rim of PEF. Normal LV fx	Normal	Normal
9	14/F	Perimyocarditis	Definite	122	Second	−0.6	Asymptomatic	hsTnI 54.9	Evidence of perimyocarditis. LVEF 60%, RVEF 54%	2 mm STE in V4-V5	Normal	Normal	Normal	Normal
10	12/M	Perimyocarditis	Definite	107	Second	8.8	Asymptomatic	TnI 14766	Evidence of myocarditis	STE in V5-V6; STD in aVR, V1; TWI in III, aVF, V4	Normal	Normal LV fx. Thin rim of PEF at systole, hyperechoic left lateral and posterior pericardium	N/A	Normal
11	17/M	Perimyocarditis	Definite	146	Second	4.3	Exercise-induced chest pain during period of exercise restriction	hsTnI 30267	Normal biV fx, (LVEF 54%, RVEF 53%) Evidence of myocarditis, LGE of epicardium, small PEF	1st degree AVB; 2 mm STE II; 2–4 mm STE in V4-V6; 2 mm STD in aVR, V1; TWI III, biphasic Ts in V3-V4	Normal	Borderline contractility, no PEF Repeat echocardiogram 7 months later: normal biV fx	N/A	Normal
12	14/M	Perimyocarditis	Definite	66	Second	1.9	Asymptomatic	TnT 646	Evidence of myocarditis. LVEF 75%, RVEF 58%	2 mm STE in V2-V5; 1 mm STD in aVR, V1	Normal	Normal	N/A	N/A
13	14/M	Myocarditis	Definite	111	First	1.1	Asymptomatic	hsTnI 184	Myocardial edema without LGE	1st degree AVB; 1 mm II,III, aVF; TWI III	Normal	Normal	N/A	N/A
14	15/M	Myocarditis	Definite	89	Second	3.8	Chest pain after playing basketball at 3 months after admission	hsTnI 263	Normal biV fx (LVEF 62%, RVEF 58%). Very mild LGE, no edema. Very mild myocarditis.	2 mm STE in V3	Normal	Normal	N/A	N/A
15	15/M	Myocarditis	Definite	104	Second	2.3	Asymptomatic	TnI 2210	Myocarditis, normal LV size and fx LVEF 65%, borderline RV fx RVEF 49%, small PEF.	2 mm STE V2-V5; 1 mm STD in aVR; TWI in aVL	2 mm STE V3-V4; 1 mm STE in II, V5-V6; Q in I, aVL	Normal	Normal	Normal
16	12/F	Myocarditis	Definite	62	Second	4.9	Occasional tachycardia and palpitations (incidental findings of goiter and hyperthyroidism treated with propranolol and carbimazole)	hsTnI 566	Mild myocarditis as evidenced by subepicardial LGE, mild subepicardial edema. Normal biV fx (LVEF 59%, RVEF 57%)	1st degree AVB; sinus tachycardia	Normal	Normal	Normal	N/A
17	14/M	Myocarditis	Definite	77	First	N/A	Occasional shortness of breath at night during sleep. No other symptoms during daytime	hsTnI 3598	Evidence of myocarditis, normal biV function	2–4 mm STE V2-V6; Biphasic Ts V3-V5	N/A	Normal	N/A	N/A
18	14/M	Myocarditis	Probable	147	First	1.0	Asymptomatic	hsTnI 514	Equivocal EGE and edema of the myocardium due to motion artefacts	Normal	Normal	Normal	Normal	Normal
19	14/M	Myocarditis	Probable	149	Second	2.3	Vasovagal near-syncope	hsTnI 201	Normal	1st degree AVB, 1 mm STD in II, III, aVF; TWI in II, III, aVF	Normal except short PR 91 ms	Normal	N/A	Normal
20	15/M	Myocarditis	Probable	161	Second	6.6	Occasional chest discomfort lasting for 5–20 min, not associated with physical activity	hsTnT 25	Depressed biV fx (LVEF 51.6%, RVEF 45.5%), no evidence of myocarditis	Normal	Normal	Normal	Normal:	N/A
21	12/F	Myocarditis	Probable	164	Second	0.5	Asymptomatic	hsTnI 141	No evidence of myocarditis. No PEF.VEF 72%, RVEF 57%	TWI in III	N/A	Mild increase LV free wall echogenicity Normal LV fx	N/A	Normal
22	13/M	Myocarditis	Probable	75	Second	1.6	Asymptomatic	hsTnT 517	T2 mild myocardial edema, no evidence of myocarditis	2 mm II in V4	Normal	N/A	Normal	N/A
23	14/M	Perimyocarditis	Probable	73	First	N/A	Asymptomatic	hsTnT 103	LVEF 56.7%, delayed subepicardial enhancement, small rim PEF	N/A	Normal	Normal	N/A	N/A
24	14/M	Myocarditis	Probable	75	Second	N/A	Asymptomatic	TnI 21678	Normal biV fx, no PEF, no evidence of myocarditis	2–5 mm STE in V3-V4; 1 mm STD aVR; TWI aVL; biphasic Ts in aVF	N/A	Normal	Normal	Normal
25	16/M	Myocarditis	Possible	72	Second	0.5	Exercise tolerance lower than before	Normal TnT (peak CRP 35.6)	No evidence of myocarditis, LVEF 66%, RVEF 58%	1st degree AVB, 2 mm STE in V2-V4	Normal	N/A	N/A	N/A
26	14/M	Myocarditis	Possible	61	Second	N/A	Asymptomatic	hsTnI 3617	No evidence of myocarditis LVEF 65.1%	Short PR, Flat T waves in III	Normal	Normal	N/A	N/A
27	17/M	Myocarditis	Possible	75	First	−7.0	Intermittent left sided chest pain both at rest and during activities	TnI 103	No evidence of myocarditis LVEF 62.9%, RVEF 56%	Early repolarization	Normal	Normal	N/A	N/A
28	12/M	Myocarditis	Possible	73	First	2.3	Asymptomatic	hsTnI 1692	N/A	Normal	Normal	Normal	N/A	N/A

AVB – atrioventricular block; biV – biventricular; BW – bodyweight; cMRI – cardiac magnetic resonance imaging; CRP – C-reactive protein; Dx – Diagnoses; EGE – early gadolinium enhancement; fx – function; hsTnI – high-sensitivity troponin I; hsTnT – high-sensitivity troponin T; LGE – late gadolinium enhancement; LV – left ventricle; LVEF – left ventricular ejection fraction; LVFS – left ventricular fractional shortening; N/A – not available; PEF – pericardial effusion; RVEF – right ventricular ejection fraction; RLPV – right lower pulmonary vein; STD – ST depression; STE – ST elevation; ST1R – Short-TI Inversion Recovery; TnI – troponin I; TnT – troponin T

*Cardiac enzymes of all patients normalized on discharge

At follow-up, 18 (64.3%) patients were asymptomatic and 10 (35.7%) had non-specific symptoms. Five patients complained of occasional chest pains or discomfort (patients 5, 11, 14, 20, and 27), two had occasional shortness of breath (patients 8 and 17), one (patient 25) had reduced exercise tolerance, and one (patient 19) had recurrent vasovagal near-syncope. All patients showed normal ECGs, echocardiograms, and treadmill ECGs that did not suggest progression to chronic heart failure. One patient (patient 16) had persistent sinus tachycardia and incidentally had goiter with hyperthyroidism unrelated to the myocarditis, which was treated with carbimazole and propranolol. At follow-up, there was a significant mean bodyweight gain of 1.81 kg (95%CI 0.47-3.1 kg, *p* = 0.01). Overall, 18 subjects (64.3%) gained between 0.5 and 8.8 kg bodyweight, whereas five subjects (17.9%) lost between 0.2 and 7 kg. Detailed physical examination and biochemical analysis, including thyroid function tests, for subjects with weight loss, were performed and did not reveal any significant pathologies.

Repeat ECGs in 27 patients performed at their referring hospitals were retrieved and reviewed. Analysis of the ECGs at the initial admission showed that 10 patients (35.7%) had sinus tachycardia (>100 bpm). No arrhythmias were observed, except for two cases of rare isolated premature ventricular contractions in one patient with definite myocarditis and one with definite perimyocarditis. Transient 1st-degree atrioventricular block was noted in one patient with perimyocarditis, but no patients developed high-grade AV block. An elevated ST segment (defined as ≥2 mm) was observed in 18 patients. A mild reduction in QRS voltage was noted in three patients with myocarditis and two with perimyocarditis. All ECGs performed during the last follow-up were mostly normal, although one patient had possible left ventricular hypertrophy and ST elevation in V3 and V4, one had ST elevation, and one had short PR intervals.

All echocardiograms reported in the electronic medical records were retrieved for review. Analysis of the echocardiograms on admission showed normal cardiac function in 16 (94.1%) out of 17 patients with myocarditis and in nine (81.8%) out of 11 patients with perimyocarditis. One patient with myocarditis and two with perimyocarditis had borderline left ventricular (LV) functions. Three patients with perimyocarditis had a small amount of pericardial effusion. One patient with perimyocarditis had incidental findings of a small coronary fistula. Of the three patients with borderline LV dysfunction (one with myocarditis and two with perimyocarditis), two had repeat echocardiograms within a week of the resolution of LV function. One patient with perimyocarditis did not receive a repeat echocardiogram at admission, although the repeat echocardiogram 7 months later was normal.

Except for one patient with possible myocarditis, cMRIs were performed on 27 patients on admission. Analysis of cMRI of patients with myocarditis (n = 16) showed one (6.23%) had borderline LV function, one (6.3%) had borderline RV function, and one (6.3%) had a borderline biventricular function. Nine patients (56.3%) also exhibited elevated extracellular volume, T2 mapping, and early and late gadolinium enhancement. Analysis of cMRI of patients with perimyocarditis (n = 11) showed three (27.3%) had borderline biventricular functions and one (9.1%) had borderline LV function. Six patients (54.5%) also had features of myocarditis, and eight (72.7%) had features of pericarditis (subepicardial or epicardial late gadolinium enhancement and pericardial effusion). Among those patients with borderline LV function, one with myocarditis and one with perimyocarditis also had echocardiograms showing borderline LV systolic function.

Due to the restrictions and limited resources during the ongoing COVID-19 outbreaks, fewer scheduled treadmill tests were available for patients. Only 16 (57.1%) out of 28 patients (7 in the myocarditis group and 9 in the perimyocarditis group) underwent treadmill testing at a median of 117 days post-admission. The results from the 16 treadmill tests showed that the patients had normal heart rate and blood pressure response, with no inducible ischemic changes or arrhythmias. However, one patient with probable myocarditis exhibited a T wave inversion in the inferior leads and anterolateral leads during the treadmill test performed two months post-admission. This patient developed perimyocarditis one month after the second dose of the BNT162b2 vaccine.

## Discussion

This is the first study to investigate the medium-term follow-up of adolescents with myocarditis and pericarditis following BNT162b2 vaccinations by an in-depth examination of clinical and cardiac outcomes. Most patients achieved complete recovery, as seen by normal serial ECGs, serial echocardiograms, and treadmill ECGs at follow-up. Only a small percentage of patients experienced chronic symptoms, including intermittent non-exertional chest pain and shortness of breath, but no specific treatment was offered as no cardiovascular pathologies could be identified. It is unlikely that these symptoms were psychosomatic, as these subjects did not complain of emotional distress after discharge or have psychiatric illnesses or mood disorders. Although echocardiograms showed most had a normal cardiac function, the use of echocardiograms alone can miss the presence of borderline ventricular function. Long-term follow-up in these patients is needed to monitor their progress.

Exercise restriction for 3–6 months is standard practice for managing patients with myocarditis and pericarditis.[16] Given that our patients only had mild symptoms, we recommended they avoid strenuous exercise for only three months, as prolonged exercise restriction may not be beneficial.[6] An unintended consequence of the exercise restriction has possibly led to many of these patients having increased bodyweight after this short period. Therefore, we recommend that patients should be given dietary and activity guidance to avoid bodyweight fluctuations, which is associated with higher all-cause mortality, cardiovascular disease morbidity, and hypertension, as demonstrated in a systematic review and meta-analysis.[17] To ensure physical fitness for the resumption of physical activities such as competitive sports, a series of cardiac assessments, including ECGs, echocardiograms, and treadmill tests, should be performed around 12 weeks after discharge.

Although the latest CDC interim recommendations suggest that subjects with a history of myocarditis or pericarditis after a dose of an mRNA vaccine should generally avoid receiving any further COVID-19 vaccines, [18] a recent population-based study conducted in Hong Kong comparing adults who had received the BNT162b2 and CoronaVac vaccines showed that the latter group had a significantly lower risk of myocarditis and pericarditis.[3] Our local study also showed that both BNT162b2 and CoronaVac vaccines are able to confer robust T cell immunity.[19] As the Hong Kong Government has approved the use of CoronaVac in children older than three years, we recommend that patients in Hong Kong who develop myocarditis and pericarditis following the BNT162b2 vaccine should receive the CoronaVac as their booster dose.

Findings from this study should be interpreted with the following caveats. First, the echocardiograms and treadmill tests were performed in different centers due to limited resources. Nevertheless, pediatric cardiologists with similar years of experience working in close collaboration performed and reported these investigations. We also acknowledge that follow-up echocardiograms and treadmill testing were unavailable for some patients as these follow-up investigations were also performed in the referring hospitals and are limited by resource availability. However, we expect the follow-up echocardiograms to be largely normal as many of these cases had a normal baseline echocardiogram even during their acute admissions. Second, due to limited resources, follow-up cMRI was not arranged as part of the follow-up protocol. However, as most subjects had mild symptoms at the initial assessments, they were expected to have normal cMRI findings at the follow-up. Third, we only recruited adolescent patients in this study. Hence, our results may not apply to adults. Last but not least, as our study sample was from the Chinese population, we cannot exclude the possibility of ethnic differences in the extent or rate of recovery in other populations. Further studies with larger sample sizes and in different populations are warranted.

## Conclusion

Adolescents with myocarditis and perimyocarditis following BNT162b2 Vaccination were recommended exercise restriction for three months, with most achieving complete recovery. The gain in bodyweight during recovery was likely due to the exercise restriction. Thus appropriate activity and dietary education should be provided at discharge. Shortening the exercise restriction period to less than three months could be considered in patients with pericarditis without myocardial involvement or if abnormal ECG findings are rapidly resolved. Long-term follow-up will be warranted for these individuals regarding their cardiovascular risk outcome. Information and advice on future vaccinations should also be incorporated into the follow-up.

## Provide Access to Data Statement

Dr Patrick Ip had full access to all the data in the study and took responsibility for the integrity of the data and the accuracy of the data analysis.
